# A New Maraging Stainless Steel with Excellent Strength–Toughness–Corrosion Synergy

**DOI:** 10.3390/ma10111293

**Published:** 2017-11-10

**Authors:** Jialong Tian, Wei Wang, M. Babar Shahzad, Wei Yan, Yiyin Shan, Zhouhua Jiang, Ke Yang

**Affiliations:** 1 School of Materials Science and Engineering, Northeastern University, Shenyang 110004, China; jltian12s@imr.ac.cn; 2 Institute of Metal Research, Chinese Academy of Sciences, Shenyang 110016, China; babar@imr.ac.cn (M.B.S.); weiyan@imr.ac.cn (W.Y.); yyshan@imr.ac.cn (Y.S.); kyang@imr.ac.cn (K.Y.); 3 School of Metallurgy, Northeastern University, Shenyang 110004, China; jiangzh@smm.neu.edu.cn

**Keywords:** maraging stainless steel, alloy design, strength and toughness, corrosion resistance, atomic probe tomography, precipitation mechanism

## Abstract

A new maraging stainless steel with superior strength–toughness–corrosion synergy has been developed based on an innovative concept of alloy design. The high strength–toughness combination is achieved by forming dispersive nano-sized intermetallic compounds in the soft lath martensitic matrix with a slight amount of residual austenite. The good corrosion resistance is guaranteed by exactly controlling the Co content based on understanding the synergistic effect between Co and Cr. The fine structure characteristics of two dominant strengthening precipitations including Ni_3_Ti and Mo-rich phases were finely characterized associated with transmission electron microscope (TEM) and atom probe tomography (APT) analyses. The relationship among microstructure, strength and toughness is discussed. The precipitation mechanism of different precipitates in the new maraging stainless steel is revealed based on the APT analysis.

## 1. Introduction

Maraging stainless steels (MSS) are a class of high strength stainless steels with excellent comprehensive performances including high strength, superior corrosion resistance and good weldability, etc. [1,2,3,4,5]. Since the first maraging stainless steel was developed by Carnegie Illionois Steel Corporation in 1946 to meet military demands, many efforts have been successfully realized to improve its performance [6,7,8,9,10,11,12,13,14]. Nowadays, new maraging stainless steel, as an important candidate material to ensure the lower energy consumptions and pollution reduction owing to its unique combination of properties including high strength, high toughness and superior corrosion resistance, is attracting much greater attention. However, a significant bottleneck that hinders the future applications of maraging stainless steels as potential structural materials is the trade-off dilemma among their strength, fracture toughness and corrosion resistance.

As far as the strength of MSS is considered, different methods have been tried to increase their strength, such as multiple-phase strengthening [1,2,3], co-precipitation of nano-scale particles [7,8], and minimal lattice misfit between matrix and precipitate [13]. Meanwhile, attentions have been concentrated on characterizing the relationship between strength and precipitate, for instances, the lattice structure, orientation relationship and evolution process during aging treatment. Besides the strength of MSS, corrosion resistance is another important and particularly critical property for future applications, because more and more materials will be subjected to extreme mechanical loads and harsh environmental conditions where corrosion is an important issue. Unfortunately, MSS with high strength is always accompanied with a loss in toughness or corrosion resistance. In general, two methods are usually considered to increase the strength of MSS. One is to add carbon (C) to strengthen the lath martensitic matrix. However, higher content of C could present a gradual incremental trend to form chromium carbides, which are harmful for both toughness and corrosion resistance [15,16]. Another method is to add more strengthening elements to increase the strength by precipitation hardening. However, most strengthening elements such as Mo, Ti and Al are also ferrite formation elements and they will promote the formation of delta-ferrite, which is harmful to the toughness. Different from those strengthening elements, Co is often added to increase the strength of maraging stainless steel by its synergistic effect with strengthening elements such as Ti and Mo [11,12,13,14]. However, Co also accelerates the spinodal decomposition of Cr atoms during the aging process, which could deteriorate the corrosion resistance [17].

In this study, a new maraging stainless steel, defined as “IMR steel” (Steel from the Institute of Metal Research), was developed based on an innovative concept of synergistic effect of multiple elements in maraging stainless steels. The microstructure of quenched IMR steel was comprised of soft lath martensitic and residual austenite. In the subsequent aging process, large amounts of nano-sized intermetallic compounds precipitate in the matrix. The IMR steel exerts a high strength in combination with high toughness and good corrosion resistance, showing outstanding performance compared with other commercial maraging stainless steels. It is expected that the new maraging steel will have potential applications in the aircraft, power generation, tools and automotive industries.

## 2. Alloy Design

According to the conventional “stainless theory”, it is generally believed that Cr must be added to above 11 wt % to impart “stainlessness” to steels. At this Cr level, an adherent and self-healing chromium oxide can be formed on the steel surface in the relatively benign corrosive environment, especially with 13 wt % Cr addition in steel, and superior corrosion resistance could be achieved [18]. Thus, the Cr content has been set as 13 wt % to guarantee a superior corrosion resistance.

Co is a key element in maraging stainless steel since it controls the balance between strength and corrosion resistance. According to previous research [17], when Co content was 13 wt %, the MSS showed a poor corrosion resistance even with 13 wt % Cr addition. When Co content was decreased to 7 wt %, the MSS showed a good corrosion resistance in 3.5% NaCl solution. Thus, the Co content has been set as 7 wt % to achieve the balance between strength and corrosion resistance.

Ti is the most effective strengthening element in maraging stainless steels. In order to reach a strength target of 1900 MPa, Ti content has been set as 1.7 wt %. Considering that the co-addition of Mo and Ti could form a core-shell structure [2] that is good for both strength and toughness, Mo content is set as 3 wt %. Although Mo is good for both strength and corrosion resistance, more Mo addition is infeasible considering the occurrence of delta-ferrite.

The role of Ni in maraging stainless steel is crucial. On the one hand, Ni could enlarge the austenite region and avoid delta-ferrite formation during cooling via austenite phase field extension. On the other hand, Ni could form the intermetallic phase, especially form η-Ni_3_Ti which is the dominant strengthening precipitate in the Ti-alloyed maraging stainless steels. Also, Ni content should be designed precisely to control the volume fraction of residual austenite that is important for both strength and toughness. Ni content in the IMR steel is designed to be 7.5 wt %.

Finally, in order to guarantee the high fracture toughness, impurities such as C, O and N should be kept less than 30 ppm, respectively. Based on the above alloy design analyses, the nominal composition of the IMR steel is designed to be (wt %) Cr 13.0%, Ni 7.5%, Co 7.0%, Mo 3.0% and Ti 1.7%.

## 3. Experimental Details

The IMR steel was first melted in a 25 kg vacuum induction melting furnace and then remelted by vacuum arc melting. Fe, Cr, Ni, Co, Mo and Ti were added in the form of high purity metals (>99.99%) to obtain a super-clean steel. Chemical composition of the steel analyzed by ICP (inductively coupled plasma) is shown in Table 1. Casting ingot with a diameter of 100 mm was homogenized at 1200 °C for 48 h and then forged into square bars (20 mm × 20 mm). Specimens for tests and microstructure characterizations were subjected to solution annealing at 1050 °C for 1 h followed by cryogenic treatment (CT) in liquid nitrogen for 8 h. Finally, aging treatment (AT) with different holding time (10 min, 0.5 h, 4 h, 16 h, 40 h, 100 h) was performed at 480 °C. Other commercial maraging stainless steels were taken from the steel enterprise and were peak aging (PA) treated to reach its highest strength. Afterwards, corrosion current density of all steels has been tested. Mechanical property (fracture toughness and ultimate tensile strength) of three steels (IMR steel, 1RK91 and Custom 475) were tested practically and other steels’ mechanical property was taken from the references. In order to guarantee the reliability, three samples have been tested to get the average.

TEM characterization was conducted using a JEM 2100 transmission microscope (Japan Electron Optical Laboratory, Tokyo, Japan) operated at 200 kV. Thin TEM foils were prepared with care for a large electron transparent area and minimum magnetic influence. 3 mm diameter discs were first mechanically polished to 50 μm and then jet electropolished in a bath containing 10 vol % HClO_4_ + 90 vol % C_2_H_5_OH at −20 °C. Needle-shaped specimens required for APT were prepared by a standard two-step electro-polishing technique. Local electrode atom probe (LEAP) acquisitions were accomplished using a LEAP™ 3000HR under an ultrahigh vacuum of ~1 × 10^−8^ Pa at a base temperature of 60 K with a pulse frequency of 200 kHz and a 20% pulse fraction. Imago Visualization and Analysis Software (IVAS) version 3.6.2 was used for data reconstruction, composition analysis and the creation of iso-concentration surface.

Tensile tests were conducted according to ASTM E8 on an AG-100KG testing machine (Shimadzu, Tokyo, Japan) at a strain rate of 2.0 × 10^−3^ s^−1^ at room temperature. Fracture toughness measurements were performed on a SCHENCK-100KN fatigue test machine (Schenck Process GmbH, Darmstadt, Germany) according to ASTM E399 at a crosshead speed of 0.5 mm/min. Potentiodynamic polarization (PDP) tests in 3.5 wt % NaCl solution were conducted using a PARSTAT4000 electrochemical workstation (Princeton Applied Research, Oak Ridge, TN, USA) with a scan rate of 0.1667 mV/s. The surface area of the working electrodes was reduced to 1.0 cm^2^ using epoxy resin, in order to avoid the edge effect. A conventional three-electrode (reference, counter and working electrodes) electrochemical cell was used in this study.

## 4. Results and Discussion

### 4.1. Microstructure and Properties

**Figure 1:**
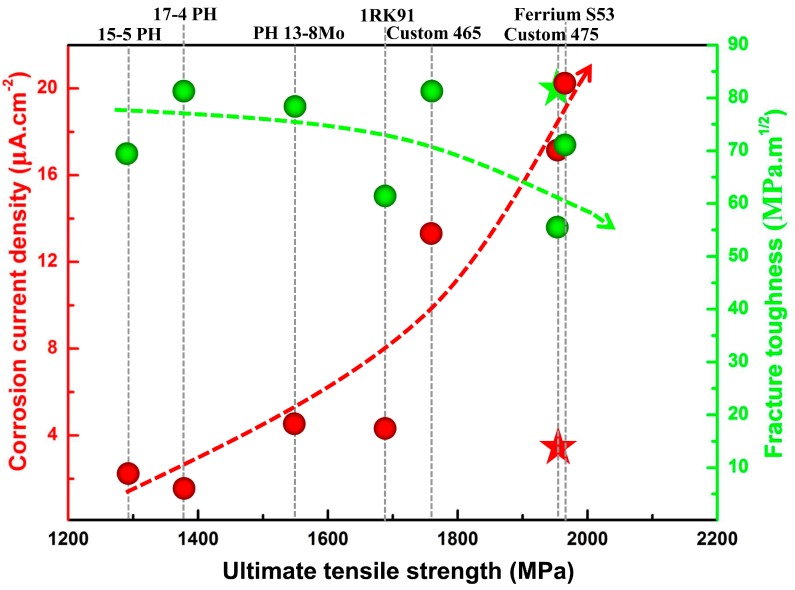
The strength–toughness–corrosion property profiles of the IMR (Institute of Metal Research) steel and comparative maraging stainless steels under peak-aged conditions. Sphere denotes the property of comparative maraging stainless steel and pentacle denotes the property of IMR steel. The mechanical property data of five commercial maraging stainless were taken from references: 15-5 PH [19], 17-4 PH [20], PH 13-8 Mo [21], Custom 465 [22], Ferrium S53 [23].

Figure 1 shows the fracture toughness, corrosion resistance and ultimate tensile strength of the IMR steel in comparison with some typical commercial maraging stainless steels. Compared with the commercial maraging stainless steels, IMR steel shows a higher ultimate tensile strength (1920 MPa) which is slightly lower than that of Custom 475 (2000 MPa). In addition to strength, two other critical properties of maraging stainless steels are fracture toughness and corrosion resistance. The corrosion current density obtained from PDP scans was used as an indicator of corrosion resistance. It can be seen clearly that 17-4 PH shows the most outstanding corrosion resistance, which could be attributed to its higher chromium content (~17 wt %). In comparison, IMR steel shows an equivalent corrosion resistance to that of 15-5 PH. Moreover, fracture toughness of IMR steel shows notable advantage compared to those maraging stainless steels. In general, strength and toughness show an inverse variation trend and it is difficult to improve both simultaneously. However, IMR steel exhibited both high toughness and superior strength, which could be attributed to its unique microstructure.

**Figure 2:**
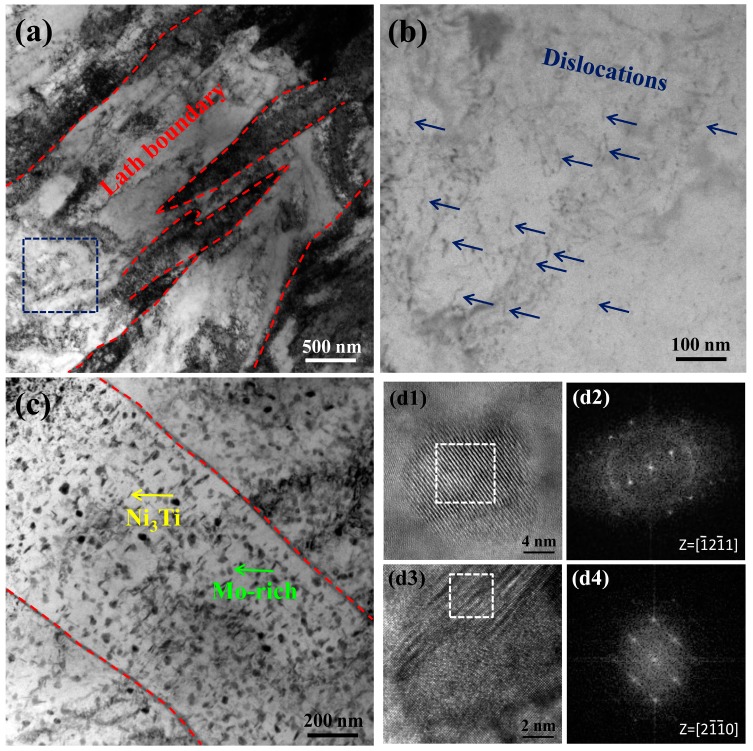
Microstructure characterization by high-resolution TEM (transmission electron microscope). (**a**) Typical martensitic lath in the specimen after CT (cryogenic treatment) treatment, lath boundary is outlined by red dashed line; (**b**) Dislocations observation in the region taken from the square in (**a**); (**c**) Morphology of Ni_3_Ti and Mo-rich precipitates in the PA-treated specimen; (**d1**) shows the high-resolution image of Mo-rich precipitate and (**d2**) shows the corresponding FFT (fast Fourier transform) pattern in the inset. (**d3**) shows the high-resolution image of Ni_3_Ti precipitate and (**d4**) shows the corresponding FFT pattern in the inset.

Figure 2a is a bright field TEM image of the specimen after cryogenic treatment (CT). Typical martensitic lath is clearly visible while high density dislocations can be found as shown in Figure 2b. The high-density dislocations in the matrix can offer preferred nucleation sites and lead to the formation of dispersive precipitates in the following aging process [24,25]. Two types of strengthening precipitates were found in the peak-aged specimen as shown in Figure 2c. According to the high-resolution TEM image (Figure 2(d1,d2)) and the corresponding FFT pattern (Figure 2(d3,d4)), the sphere-like precipitates and rod-like precipitates are identified as Mo-rich phase and Ni_3_Ti phase, respectively.

**Figure 3:**
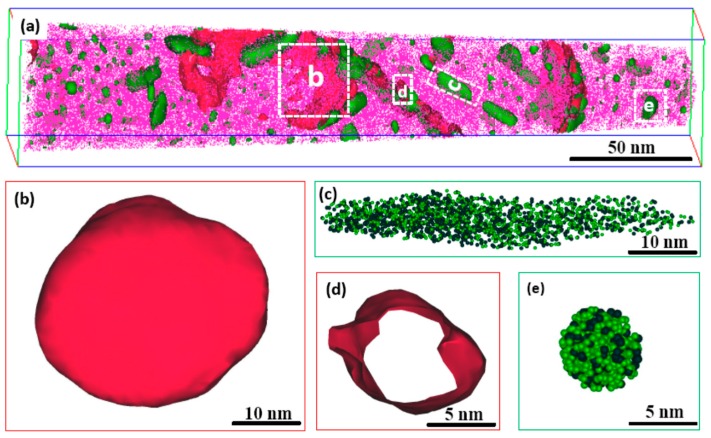
Morphology of precipitates observed by atom probe tomography (APT) analysis in PA-treated specimen. (**a**) 3-D reconstruction of the atomic positions of Fe (**pink points**), isoconcentration surface for regions containing more than 10 at % Mo (**red surfaces**) and 35 at % Ni + Ti (green surfaces); (**b**) Sphere-like Mo-rich phase outlined by 10 at % Mo isoconcentration surface; (**c**) Rod-like Ni_3_Ti phase outlined by Ni (**green**) and Ti (**grey**) atoms; (**d**) Flake-like Mo-rich phase outlined by 10 at % Mo isoconcentration surface; (**e**) Sphere-like Ni_3_Ti phase outlined by Ni (**green**) and Ti(**grey**) atoms.

**Figure 4:**
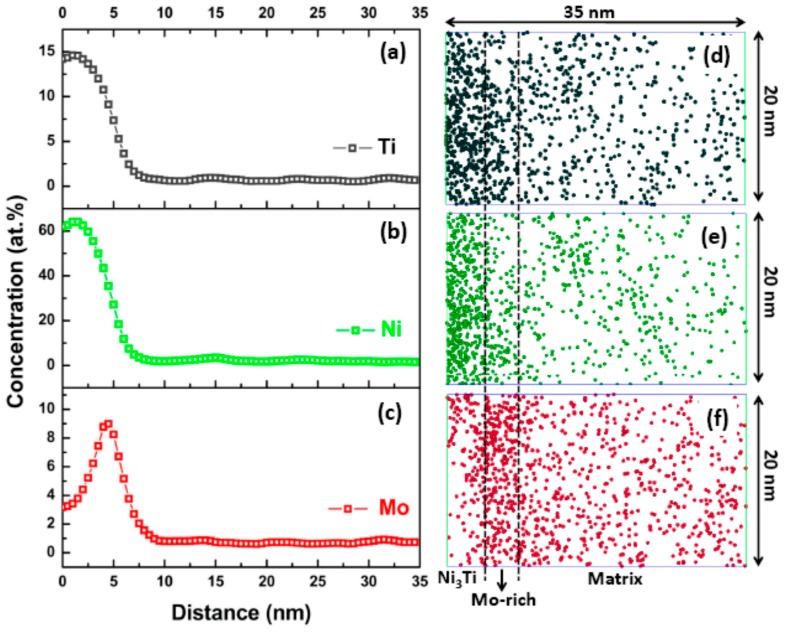
1-D reconstruction of the atomic positions (**d**–**f**), and the corresponding one-dimensional concentration profile (**a**–**c**), across the Ni_3_Ti/matrix interface for peak-aged specimen.

As a powerful technique to characterize the nano-sized precipitates, atom probe tomography (APT) is characteristic for its near-atomic spatial resolution and can offer the information of the precipitations such as composition, spatial morphology, size, number density, etc., which is beyond the scope of TEM analysis. In this study, regions that contain more than 10 at % Mo and 35 at % Ni + Ti are defined as Mo-rich phase and Ni_3_Ti phase, respectively. Figure 3a shows the 3-D reconstruction of the atomic positions of Fe (pink points), isoconcentration surface for regions containing more than 10 at % Mo (red surfaces) and 35 at % Ni + Ti (green surfaces). A careful observation of two types of precipitates revealed that each precipitate has two different shapes, sphere-like Mo-rich phase and bar-like Ni_3_Ti phase, as shown in Figure 3b,c, which were also observed by TEM. Meanwhile, Mo-rich phase and Ni_3_Ti phase have another two refined structures which were not observed by TEM. As shown in Figure 3d,e, a ring-like Mo-rich phase and sphere-like Ni_3_Ti phase can be observed. It is believed that small sphere-like Ni_3_Ti phases were formed at the beginning stage of aging treatment and, due to lack of time or hindrance of phase interface, they could not grow to big rod-like precipitates. On the contrary, those with the minimal interface energy between Ni_3_Ti and matrix grew and finally formed rod-like Ni_3_Ti precipitates. In case of Mo-rich phase, except for sphere-like precipitates, ring-like precipitates were also observed located at the interface between Ni_3_Ti and matrix. The corresponding 1-D concentration profile and atomic maps within a selected region crossing the Ni_3_Ti/matrix interface are shown in Figure 4. The region between the two dashed lines corresponds to the Mo-rich phase with thickness of about 5 nm. The results indicate that Mo-rich phase is more likely to nucleate at the Ni_3_Ti/matrix interface, and its forming mechanism will be discussed later.

The good combination of strength and toughness for the IMR steel can be attributed to the following two aspects. First, since Ti precipitates most rapidly, strongly and completely during aging in the maraging stainless steel [26], Ni_3_Ti precipitates make a dominant contribution to the strength. Meanwhile, Mo-rich phase retards the growth of Ni_3_Ti precipitates through its accumulation at the interface [1]. Thus, Mo-rich phase could promote the maintenance of highly dispersive Ni_3_Ti nano-precipitates and enhance the strengthening effect of Ni_3_Ti precipitates. Second, the highly uniform distribution of Ni_3_Ti precipitates effectively reduces stress concentration. In addition, Mo-rich phase at the interface results in a small lattice misfit between Ni_3_Ti and matrix [1]. Thus, the associated elastic interaction between precipitate and dislocation should be lowered. Therefore, the core-shell structure effectively prevents the crack initiation at the precipitate-matrix interface owing to the negligible strain accumulation and thus guarantees a good combination of strength and toughness.

### 4.2. Precipitation Mechanism of Ni_3_Ti and Mo-Rich Precipitates

**Figure 5:**
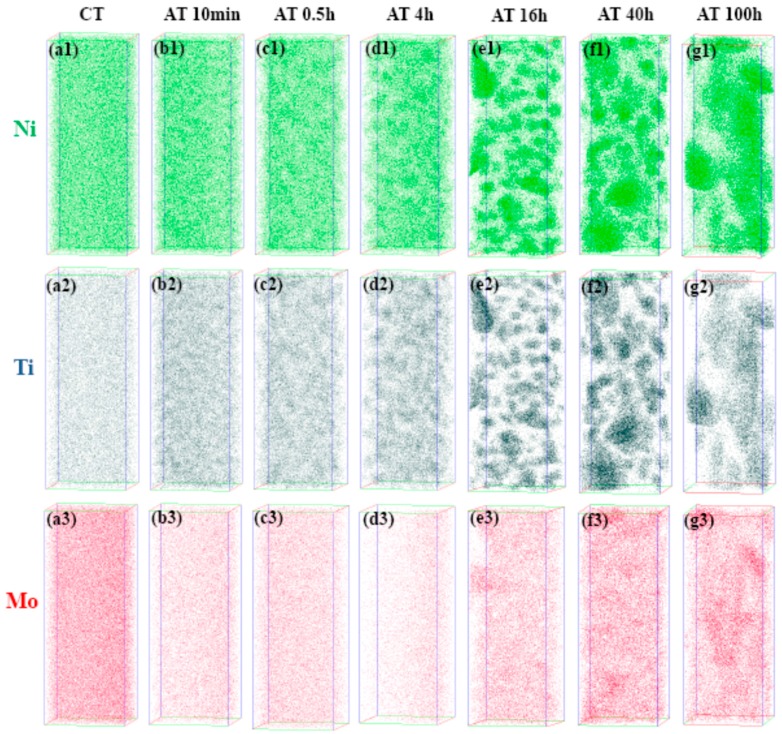
Three-dimensional reconstruction of the atomic positions of Ni (**green points**), Ti (**grey points**) and Mo (**red points**) for specimens aged at 753 K for different time. All the analyzed volumes are with the dimension of 30 × 30 × 80 nm^3^.

APT was used to further characterize the evolution of three elements (Ni, Ti, Mo) during the aging treatment, and Figure 5 shows the visual atomic position reconstruction. It can be seen clearly that Ni and Ti show similar distribution positions, which indicates that Ni and Ti form clusters together whereas Mo forms cluster separately. Maximum separation envelope methodology (MSEM) was used to further analyze the precipitate kinetics of different clusters. In this method, the spherical volume region around the selected atoms, as defined by a maximum separation distance, d_max_, is searched for any other selected solute atoms. The minimum solute atoms number (N_min_) threshold parameter was used to limit the size of the solute clusters to those more than the threshold value. The cluster numbers were counted based on specific parameters (d_max_ and N_min_) and the cluster density was defined as the ratio of cluster number over the volume of analyzed body. Table 2 shows the statistical results about the clusters density under different aging condition based on the MSEM.

Both cluster analysis results in Table 2 and atomic positions reconstruction in Figure 5a indicate that no cluster exists in the steel matrix after CT. When aging time was 10 min, Ni and Ti clusters occurred as shown in Figure 5(b1,b2), and the cluster density of Ni + Ti reached 59.92 × 10^−5^ nm^−3^ as shown in Table 2. However, no Mo cluster formed after 10 min according to the cluster analysis results. Cluster density of Ni + Ti cluster reached to maximum (116.78 × 10^−5^ nm^−3^) after aging for 0.5 h, and with increase of aging time, cluster density decreased slightly as a result of the aggregation and growth of Ni_3_Ti precipitates. According to the cluster analysis results of Mo in Table 2, it can be found that Mo cluster density only reached to 0.26 × 10^−5^ nm^−3^ even after aging for 4 h. Meanwhile, no obvious cluster was observed in the atomic reconstruction (Figure 5(d3)). The maximum value of Mo cluster density was 51.52 × 10^−5^ nm^−3^ after aging for 16 h.

The results indicate that Ni and Ti showed much quicker aging response than Mo at the early aging stage. Considering the diffusion coefficients of the portioning elements (Ni, Ti, Mo, Fe) in the α-Fe matrix [27,28,29], the diffusivity of Mo is much lower than that of the other elements. This indicates that Mo acts as a kinetically controlling element for the precipitation process, as revealed by the fact that Mo is rejected from the precipitate into the matrix, accumulating before the Ni_3_Ti/matrix interface.

**Figure 6:**
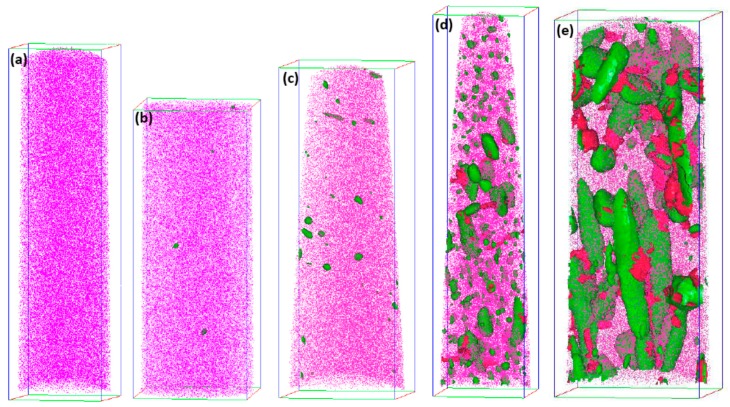
Three-dimensional reconstruction of the atomic positions of Fe (**purple pints**) and isoconcentration surface for regions containing more than 35 at % Ni + Ti (**green surfaces**) and 5 at % Mo (**red surfaces**) for specimens (**a**) aged for 10 min; (**b**) aged for 0.5 h; (**c**) aged for 4 h; (**d**) aged for 40 h; (**e**) aged for 100 h. Bounding box size: (**a**) 58 × 59 × 218 nm^3^; (**b**) 55 × 55 × 144 nm^3^; (**c**) 64 × 65 × 182 nm^3^; (**d**) 59 × 60 × 247 nm^3^; (**e**) 67 × 69 × 173 nm^3^.

**Figure 7:**
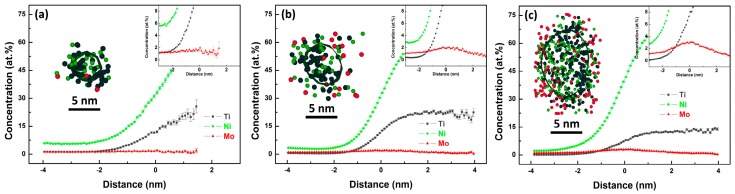
Proximity histograms of Ni_3_Ti precipitate in different specimens (**a**) aged for 4 h; (**b**) aged for 16 h; (**c**) aged for 100 h. The inserts show the 1 nm thick atom maps through the centers of representative Ni_3_Ti precipitate in specimens under different aging conditions (Ni for green sphere, Ti for grey sphere, Mo for red sphere).

Figure 6 shows the distribution characteristics of Ni_3_Ti phase outlined by green Ni + Ti 35 at % isoconcentration surface and Mo-rich phase outlined by red Mo 5 at % isoconcentration surface at different aging stages. When the aging time was 0.5 h, sphere-like Ni_3_Ti particles are evident, as shown in Figure 6b. When the aging time extended, a further growth of particles seems to occur, and their average diameter also increases. But the evolution of Mo-rich phase was different, as no Mo 5 at % isoconcentration surface was detected in the specimen aged for 4 h, as shown in Figure 6c, which could be attributed to a low Mo concentration at Ni_3_Ti/matrix interface. According to the proximity histogram analysis shown in Figure 7a, the Mo concentration at Ni_3_Ti/matrix interface approximates to the average concentration of Mo in the matrix (1.58 at %). When aging time prolonged to 40 h (Figure 7b) and 100 h (Figure 7c), the Mo concentration at Ni_3_Ti/matrix interface increases to 2.09 at % and 3.06 at %, respectively.

Since the diffusivity of Mo is much lower than those of Ni and Ti, this indicates that Ni_3_Ti nucleates headmost and will seize most prior nucleation sites such as grain boundary, blocky/lath boundary and dislocation. Considering that these defects are also favorable diffusion paths, a low nucleation rate of Mo-rich phase can be predicted. This well explains the larger size and lower number density Mo-rich phase. As shown in Figure 3a, the dimension of sphere-like Mo-rich phase is almost similar to the cross section (~50 × 50 nm^2^) of the APT analytical body. Attributed to the size limitation of APT analytical body, sphere-like Mo-rich phase was not included in the analytical body, as shown in Figure 6d,e. This also results in an anomaly phenomenon in Table 2 that Mo cluster density had a sharp decrease after aging time reached to 40 h and 100 h.

**Figure 8:**
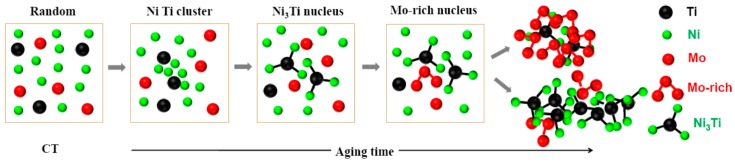
Schematics showing precipitation mechanism of Ni_3_Ti and Mo-rich phases in the new maraging stainless steel.

Based on the above characterization on the formation and evolution of the Ni_3_Ti and Mo-rich phases, the precipitation mechanism in the new maraging stainless steel can be revealed as follows and schematically illustrated in Figure 8. At the initial stage of aging, Ni and Ti firstly precipitate out of the supersaturated solid solution to form Ni + Ti cluster together. During the rapid initial growth process of Ni_3_Ti, Mo solutes are rejected from Ni_3_Ti and finally segregate at the Ni_3_Ti/matrix interface. Since the precipitation of Ni_3_Ti has seized most prior nucleation sites and favorable diffusion paths, such as grain boundary, blocky/lath boundary and dislocation, Mo-rich phase is inclined to nucleate at the Ni_3_Ti/matrix interface as a result of Mo atoms accumulation. After stable Mo-rich phase nucleation occurs, considering that the diffusion of Mo is difficult, most nuclei grow up slightly to form final flake-like Mo-rich phase adjacent to Ni_3_Ti phase. Thus, the flake-like Mo-rich phase and Ni_3_Ti phase form a core-shell structure together. In different sites, portion of nucleus grow up drastically to form large size sphere-like Mo-rich phase and even build a crosslink to each other.

## 5. Conclusions

This study presents an understanding of the mechanism of the evolution of precipitation in a new maraging stainless steel. The features of different kind of precipitations are carefully characterized at atomic scale based on combination of APT and TEM analyses. The following conclusions are drawn from the study:

A new maraging stainless steel with high strength (above 1920 MPa), high toughness (~80 MPa·m^1/2^) and superior corrosion resistance (comparable to 15-5 PH) was developed. The new maraging stainless steel is strengthened by two dominant strengthening precipitations including Ni_3_Ti and Mo-rich phases. Different morphologies of these two strengthening precipitates (rod-like and sphere-like Ni_3_Ti phase, flake-like and sphere-like Mo-rich phase) were observed by APT.

In the aging process, Ni and Ti solutes show a much quicker precipitation response than Mo solute. Associated with morphology observation and cluster analysis, the precipitation mechanism is succinctly proposed as: supersaturated solid solution → Ni + Ti cluster → Ni_3_Ti nucleus → Ni_3_Ti nucleus and Mo-rich segregation → core-shell structure (Ni_3_Ti phase and flake-like Mo-rich phase) or large sphere-like Mo-rich phase depending on the grow up process of Mo-rich nucleus.

## Figures and Tables

**Table 1:** Chemical composition of Institute of Metal Research (IMR) steel (wt %).

Cr	Ni	Co	Mo	Ti	C	O	N	Fe
12.53	7.45	7.16	3.14	1.75	0.0024	0.0028	0.0026	Bal.

**Table 2:** Cluster analysis of specimens under different aging conditions. (Ni + Ti cluster: d_max_ = 0.5, N_min_ = 100; Mo cluster: d_max_ = 0.5, N_min_ = 10).

Items	CT	AT 10 min	AT 0.5 h	AT 4 h	AT 16 h	AT 40 h	AT 100 h
Volume of analyzed body (nm^3^)	638,160	745,996	554,895	757,120	772,475	874,380	799,779
Ni + Ti cluster density (10^−5^ nm^−3^)	0	59.92	116.78	114.51	79.74	56.04	15.63
Mo cluster density (10^−5^ nm^−3^)	0	0	0	0.26	51.52	3.09	9.38
